# Mesenchymal Bmp7 Controls Onset of Tooth Mineralization: A Novel Way to Regulate Molar Cusp Shape

**DOI:** 10.3389/fphys.2020.00698

**Published:** 2020-07-03

**Authors:** Zeba Malik, Daniela M. Roth, Farah Eaton, Jessica M. Theodor, Daniel Graf

**Affiliations:** ^1^School of Dentistry, Faculty of Medicine and Dentistry, University of Alberta, Edmonton, AB, Canada; ^2^Department of Biological Sciences, University of Calgary, Calgary, AB, Canada; ^3^Department of Medical Genetics, Faculty of Medicine and Dentistry, University of Alberta, Edmonton, AB, Canada

**Keywords:** tooth mineralization, bone morphogenetic protein, signaling, epithelial–mesenchymal interaction, mouse genetics, gene expression

## Abstract

Investigating the molecular basis for tooth shape variation provides an important glimpse into the evolution of tooth function. We recently showed that loss of mesenchymal BMP7 is sufficient to alter morphology and function of the toothrow. Here we report on the underlying mechanism. Expression of mesenchymal Bmp7 is observed at sites where mineralization is initiated, in tooth cusps of developing molars. Neural crest-specific deletion of *Bmp7* (Bmp7^ncko^) resulted in a complete lack of dentin/enamel formation at birth, the time when mineralization is normally initiated in the upper molars, similar to what was observed in Bmp2^ncko^ mice. Unlike loss of *Bmp2*, loss of *Bmp7* did not affect odontoblast polarization and did not significantly alter the levels of pSmad1/5/8, but almost completely abolished canonical Wnt signaling in (pre)-ameloblasts. Tooth mineralization resumed with a 48-h delay allowing for additional mesenchymal proliferation. Enamel volume was still reduced at P4 and P8, but was comparable in erupted teeth, which were broader and had altered cusp shapes. Tooth eruption was also delayed. Overall, enamel appeared inconspicuous, although some structural changes along with reduced mineral density could be observed. Loss of Bmp7 led to an increase in mesenchymal Bmp6 suggesting an interplay between Bmp6 and Bmp7 in the regulation of mineralization initiation. Our findings show that regulation of the onset of tooth mineralization is a hitherto unsuspected mechanism controlling tooth shape variation. Initiation of tooth mineralization is regulated by a complex epithelial-mesenchymal Bmp/Wnt-signaling network to which Bmp7 contributes. This network is separate and independent of the Bmp2-signaling network regulating odontoblast cell polarization. From an evolutionary perspective, addition of Bmp7 as initiator of tooth mineralization might be akin to an upgrade of an existing computer operating system. While not essential, it provides obviously sufficient advantage warranting its evolutionary incorporation.

## Introduction

Enamel and dentin formation, the two major mineralized compartments of the tooth, are regulated by intricate signaling events between the different hard tissue-forming cells, ameloblasts and odontoblasts. Although changes to these signaling events may result in disturbed or poor quality mineralized structures (Kim and Simmer, 2007; Coxon et al., 2012), dental hard tissue formation is remarkably robust. Patterning of molar cusps precedes their mineralization, which is initiated at the tip of a cusp (Simmer et al., 2010). Variations in mineralization and molar cusp shape contribute to dental diversity and functionality and thus provide important parameters for understanding ecological processes that drive evolution (Jernvall and Jung, 2000; Salazar-Ciudad and Jernvall, 2002; Kavanagh et al., 2007; Ungar, 2009).

The outer dentin surface is specified by the interface between the epithelium and mesenchyme which becomes the dentino–enamel junction in developing crown. Enamel crown formation on the outer dentin surface starts with the onset of biomineralization at the future cusp tips defined by growth parameters (Salazar-Ciudad and Jernvall, 2002, 2010; Simmer et al., 2010). A better understanding of how crown growth is regulated would be essential in defining evolutionary crown morphology in hominoids (Dean and Reid, 2001). Epithelial enamel knots direct folding of the inner enamel epithelium by producing diffusible molecules that inhibit new knots nearby and initiate them only in the zones outside the previously initiated cusps (Jernvall, 2000). The primary enamel knot occurs at the tip of the first cusp and directs the formation of secondary or tertiary enamel knots before the tooth starts to mineralize (Jernvall, 2000; Luukko et al., 2003). Studies over the past two decades have focused on enamel knot activation and silencing in defining multicuspid molar formation.

Members of the bone morphogenetic protein (Bmp) family are evolutionarily conserved signaling molecules belonging to the Tgfβ family. They can be grouped into several subfamilies and might function as homo- or heterodimers to control a multitude of developmental processes including craniofacial development (Graf et al., 2016; Kim et al., 2019; Wisotzkey and Newfeld, 2020). Various Bmps are expressed at all stages of tooth development (Aberg et al., 1997). They are found in enamel knots that control cusp formation and are critical components of reciprocal epithelial–mesenchymal signaling events that coordinate early tooth mineralization (Wang et al., 2004; Malik et al., 2018; Meguro et al., 2019). For instance, Bmp2 is expressed in odontoblasts at onset of tooth mineralization, where it coordinates odontoblast polarization to ensure ordered dentin deposition. Loss of mesenchymal Bmp2 results in a dentinogenesis imperfecta-like phenotype (Malik et al., 2018). Various Bmps are often expressed in close vicinity, lending support to the notion that they might be redundant or function as heterodimers. In addition to Bmp2, Bmp7 is also expressed in the cusp and in odontoblasts at the onset of tooth mineralization (Aberg et al., 1997). However, neural crest-specific deletion of *Bmp7*, which includes mesenchymal dental pulp cells, leads to the formation of wider teeth and the appearance of extra cusps with no apparent effect on mineralization (Zurowski et al., 2018). These changes in tooth morphology have functional consequences and implications for mammalian dental evolution (Zurowski et al., 2018). These divergent phenotypes suggest that Bmp2 and Bmp7, although likely expressed in close spatial and temporal vicinity, act largely independent. Here we use early tooth mineralization as a unique system to address redundancy and independence of these two Bmps. We find that loss of Bmp7 results in a delay of tooth mineralization and tooth eruption. We explore the molecular/cellular mechanisms that underlie this mineralization delay as well as the formation of wider/extra cusps in Bmp7-deficient molars. We find that these independent roles of Bmp2 and Bmp7 are characterized by the differential use of downstream signaling pathways. Whereas Bmp2 coordinates tooth mineralization, Bmp7 acts to initiate tooth mineralization.

## Materials and Methods

### Animals

Animal experiments were approved by the Research Ethics office of the University of Alberta (Animal Use and Care Committee protocol AUP1149) in compliance with guidelines by the Canadian Council of Animal Care. Mouse lines were backcrossed more than 10 generations to the C57Bl/6J background. Bmp7^fl/fl^ mice (Zouvelou et al., 2009a) were crossed to Wnt1-Cre mice [Tg(Wnt1-cre)11Rth] for neural crest-specific deletion of *Bmp7* (subsequently referred to as Bmp7^ncko^). *Bmp7* expression was detected using Bmp7lacZ reporter mice (Godin et al., 1998). Mice were PCR-genotyped with DNA obtained from tissue biopsies as described (Zouvelou et al., 2009b; Segklia et al., 2012).

### Micro-Computed Tomography (μCT) Analysis

MicroCT scans were obtained using a MILabs μCT (Milabs, Utrecht, Netherlands) at the School of Dentistry, University of Alberta. For live scanning, mice were anesthetized using isoflurane. For scans from post-mortem, dissected tissues, samples were fixed in 4% paraformaldehyde (PFA) for 24 h, washed, and stored in PBS prior to scanning. Scans were acquired in a mouse bed holder using the ultra-focused setting with following parameters: voxel size = 10 μm; voltage = 50 kV; current = 0.24 mA; and exposure time = 75 ms. Scans were reconstructed at a voxel size of 25 μm or smaller and analyzed using the AVIZO 3D software (Life Technologies, version 2019.1). To determine the total mineral volume of enamel or dentin, a manual segmentation was performed using appropriate gray level values corresponding to the single mineralized tissues (enamel, dentin, bone). Mineral density was determined using Hounsfield units.

### Tissue Preparation and Histology

Control and mutant embryonic heads or isolated adult mandibles were fixed in 4% PFA. Samples were decalcified using 0.5M EDTA solution for 1 day (newborn heads) to 4 weeks before processing for paraffin embedding. Paraffin blocks were cut on a type ‘820’ Spencer microtome at 5–7 microns and stored at room temperature until use. For histological analysis, sections were placed in an oven at 60°C for 30 min, de-paraffinized in xylol, rehydrated in a decreasing ethanol gradient followed by staining with hematoxylin and eosin (H&E), immunohistochemistry (IHC), or immunofluorescence (IF).

### Immunohistochemistry (IHC) and Immunofluorescence (IF)

Tissue sections were boiled in 10 mM sodium citrate buffer (pH 6) for 1 min in a microwave and allowed to cool to room temperature to facilitate antigen retrieval. Sections were blocked with 1% BSA + 0.5% Tween in PBS. Primary antibodies were incubated overnight at 4°C in blocking solution in a humidified chamber. Details of primary and secondary antibodies and dilutions used are summarized in Supplementary Table S1.

### LacZ Staining

LacZ staining was performed as described previously (Zouvelou et al., 2009a). Alternatively, mice were directly perfused with lacZ staining solution; mandibles were dissected, stained, fixed, and processed as described above.

### Scanning Electron Microscopy (SEM)

For backscatter scanning electron microscopy (SEM) imaging, mandibles were dissected, fixed in 4% PFA for 2 days, washed, and dehydrated in series of ascending grades of alcohol and embedded in sagittal orientation in Technovit 7200 VLC. Sections were prepared for imaging using a cutting and grinding system (Wolff et al., 2010). Processing of samples and imaging was performed by electron microscopic facility staff at the Institute of Oral Biology, University of Zurich.

### RNA Extraction and Quantitative Real-Time PCR (qRT-PCR)

Tooth germs from newborn (postnatal day 0, P0) control or Bmp7^ncko^ pups were dissected and immediately processed for extraction of total RNA using Thermo Scientific RNA extraction kit (#K0731). cDNA was transcribed from 0.5 to 1 μg RNA using Thermo Scientific cDNA kit (#K1620). Quantitative RT-PCR was performed with 5–10 ng cDNA/reaction using appropriate primer pairs (see Supplementary Table S2) and a SYBR green-based amplification kit (SsoAdvanced^TM^ Universal SYBR^®^ Green Supermix-Biorad) on a BioRad C1000 Touch Thermal Cycler. Relative expression was determined in relation to the housekeeping gene 36B4 using the ddCt method (Schmittgen et al., 2000). Analysis was performed on triplicate samples, and data shown are representative from at least three independent biological repeats.

### Statistical Analysis

RT-PCR and quantitative data are presented as mean ± SD. Analysis between groups was performed using an unpaired Student’s *t*-test using Microsoft Excel. A *P*-value < 0.01 was considered to be statistically significant. For RT-qPCR analysis, groups were performed in triplicates. Number of biological repeats of each independent experiment is denoted as *n*.

## Results

### *Bmp7* Is Expressed in Differentiating Odontoblasts and a Subset of Ameloblasts at Early Mineralization Stages

**FIGURE 1:**
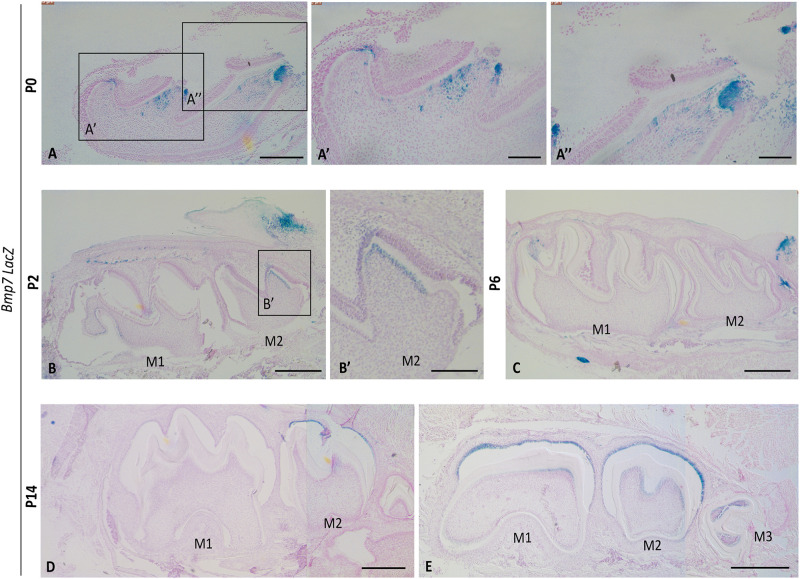
*Bmp7* expression in odontoblasts correlates with onset of mineralization. Sagittal sections of molars from *Bmp7*LacZ reporter mice **(A)** at P0 Bmp7 is expressed in a subset of odontoblasts at the crown region in the cusps of the 1st maxillary molar. **(A’,A”)** Magnified view of **(A)**. **(B)** At P2 *Bmp7* expression is restricted to the 2nd molar and is not visibly noticed in 1st mandibular molars. **(B’)** Magnified view of 2nd molars shown in **(B)**. **(C)** At P6 expression is lost in both 1st and 2nd molars. **(D,E)** At P14 *Bmp7* expression is dynamic and Bmp7 appears in the 3rd molar and is re-expressed in some lateral areas of the 1st/2nd molars. Expression of Bmp7 in ameloblasts is dynamic and can be observed at various stages. Scale bars: **(A–E)**: 250 μm, **(A’)**: 100 μm, **(A”,B’)**: 50 μm.

Previous studies reported that Bmp7 is expressed in dental epithelium (placode, enamel knot) at early stages of tooth development, but shifts to the dental mesenchyme around the time tooth mineralization is initiated (Aberg et al., 1997; Helder et al., 1998; Zouvelou et al., 2009b). We confirmed this shift using *Bmp7*LacZ reporter mice. At E16.5, *Bmp7* expression was restricted to the enamel knot in the epithelium, and at E18.5, expression in both mesenchyme and epithelium was hardly detectable (Supplementary Figure S1). At P0, when the upper first molar is in its early mineralization stages, *Bmp7* expression was restricted to differentiating odontoblasts at the tip of cusps or mesenchymal cells in their vicinity (Figures 1A,A’). Expression was also observed in a subset of ameloblasts in vicinity to *Bmp7*-positive odontoblasts (Figures 1A,A”). This mesenchymal expression in the 1^st^ molar was variable and transient and was not observed at P2. At this stage, *Bmp7* expression was noted in the sequentially differentiating odontoblasts of the 2nd molar (Figures 1B,B’). To note, ameloblasts expressing *Bmp7* were found at the non-secretory side of the cusp evident by lack of mineral matrix and flattened morphology (Figure 1B’ arrows). At P6, no *Bmp7* expression was observed in odontoblasts (Figure 1C). At P14, expression was variable (Figures 1D,E), with expression being noted in odontoblasts in more lateral regions of the 1st/2nd molar and the 3rd molar.

### Neural Crest-Specific Deletion of Bmp7 Results in Delayed Tooth Mineralization

**FIGURE 2:**
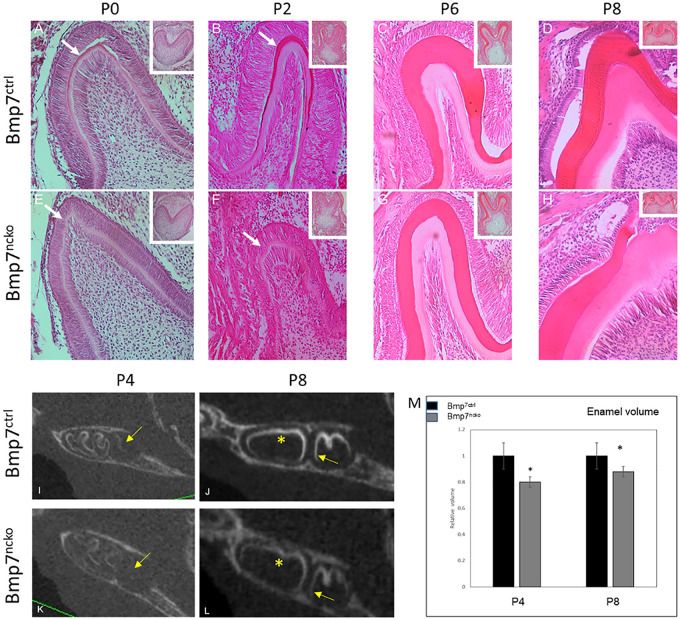
Loss of mesenchymal Bmp7 leads to a delay in tooth mineralization. **(A–H)** H&E stained frontal sections from 1st mandibular molars of control **(A–D)** and Bmp7^ncko^ mutant mice **(E–H)** at P0 **(A,E)**, P2 **(B,F)**, P6 **(C,G)**, and P8 **(D,H)** show an initial delay of onset of tooth mineralization. At P0, maturation and organization of ameloblasts and odontoblasts appears delayed with little or no deposition of mineral matrix. At later stages dentin and enamel are formed. **(I–L)** Sagittal ortho-slices from microCT scans at P4 **(I,K)** and P8 **(J,L)** of control **(I–J)** and Bmp7^ncko^ mutant mice **(K,L)** showing delayed mineralization. Note lack of mineralization at P4 and delayed mineralization at P8 in the 2nd molar in Bmp7^ncko^ (arrows) and reduced mineral density (star). **(M)** Quantification of tooth mineralization from microCT scans at P4 and P8 show that differences persist until at least P8. ^∗^*p* < = 0.05.

Deletion of *Bmp7* in the dental mesenchyme resulted in a lack of mineralization at P0, similar to what is seen with the deletion of *Bmp2* (Malik et al., 2018). Odontoblasts and ameloblasts appeared less mature, and in particular, ameloblasts were less polarized. Hardly any mineral matrix deposition was evident when compared to control molar cusp (Figures 2A,E, arrows). At P2, the dentin and enamel mineral matrix deposition had progressed in molar cusps of control mice (Figure 2B, arrow). Mice lacking Bmp7 still showed little evidence of mineral matrix deposition (Figure 2F, arrow). However, by P6, mineralization could be observed in mutant teeth. Polarized ameloblasts and odontoblasts were evident along with a defined dentin and enamel matrix (Figures 2C,G). Overall appearance was fairly similar to control molars. At P8, maturation of the mineral matrix progressed in both control and mutant molars and a distinction between pre-dentin and mature dentin was evident (Figures 2D,H). To assess whether mineralization was objectively reduced at P8, we performed volume quantification of the mineralized tooth structures from microCT scans at P4 and P8. The volume was reduced in the mutant at both stages. Furthermore, mutant enamel showed reduced radiolucency at P8 (Figures 2I–M). These findings indicate that Bmp7 is involved in regulating the onset of tooth mineralization.

**FIGURE 3:**
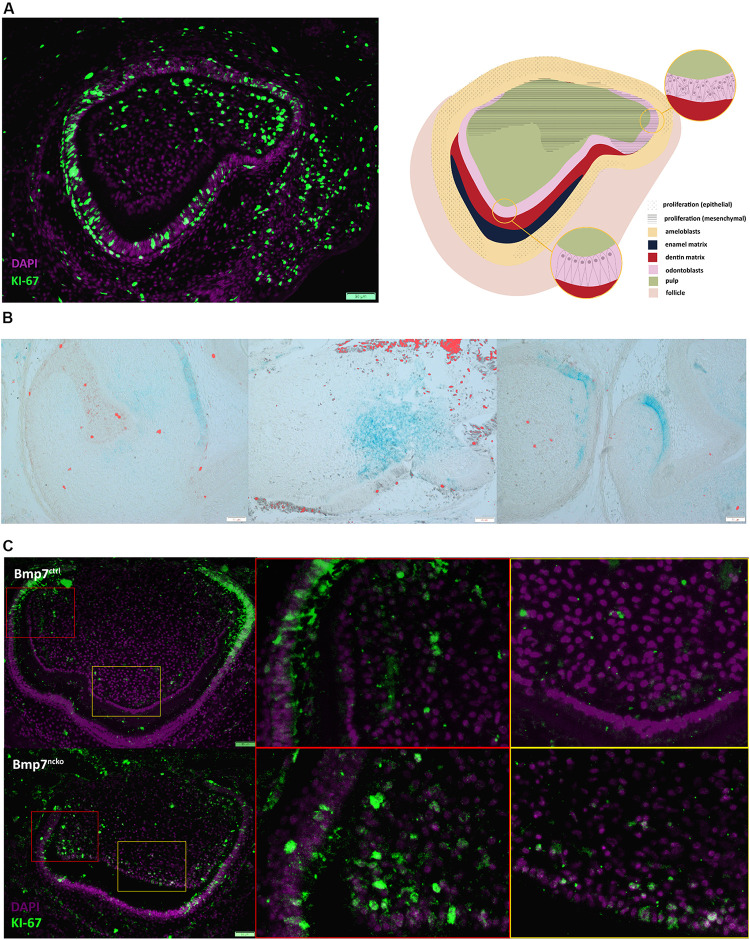
*Bmp7* expression is associated with termination of proliferation. **(A)** (left panel) Frontal section of a P0 molar stained for Ki67 (green) to identify cell proliferation counterstained with DAPI (purple). Note mesenchymal proliferation has ceased in the cusp with polarized odontoblasts, whereas mesenchymal proliferation continues in the more immature, lateral cusp. (Right panel) Schematic representation of panel to the left. **(B)** *Bmp7*LacZ staining in relation to Ki67 indicates that areas with mesenchymal Bmp7 are non-proliferating. **(C)** Frontal sections of P0 Bmp7 control or mutant molars showing that loss of Bmp7 is associated with persistent mesenchymal proliferation in the cusp area including odontoblasts (orange boxes) and loss of epithelial proliferation toward cervical (red boxes). Scale bars: 50 μm.

Reduction or loss of Bmp7 results in slightly enlarged, broader teeth (Saito et al., 2016; Zurowski et al., 2018). To explore if the delay in mineralization initiation resulted in additional cell proliferation in the cusp areas, we assessed cell proliferation in relation to mesenchymal Bmp7-lacZ expression. In a P0 wild-type control tooth, mesenchymal proliferation had ceased in cusp areas showing morphological features consistent with the onset of mineralization (odontoblast polarization), but continued in less mature, lateral cusp areas (Figure 3A). Correlating proliferation to *Bmp7* expression, areas showing Bmp7-lacZ staining were generally devoid of proliferating cells, whereas proliferation could be detected in adjacent lacZ-negative areas (Figure 3B). Deletion of mesenchymal Bmp7 resulted in persistent mesenchymal proliferation in areas where mineralization was delayed at P0 (Figure 3C). This suggests that delaying mineralization allows for additional mesenchymal proliferation expected to affect overall tooth size and leading to minor cusp shape variations, as observed in mice deficient for mesenchymal *Bmp7* (Zurowski et al., 2018).

### Bmp7 Controls Onset of Tooth Mineralization

**FIGURE 4:**
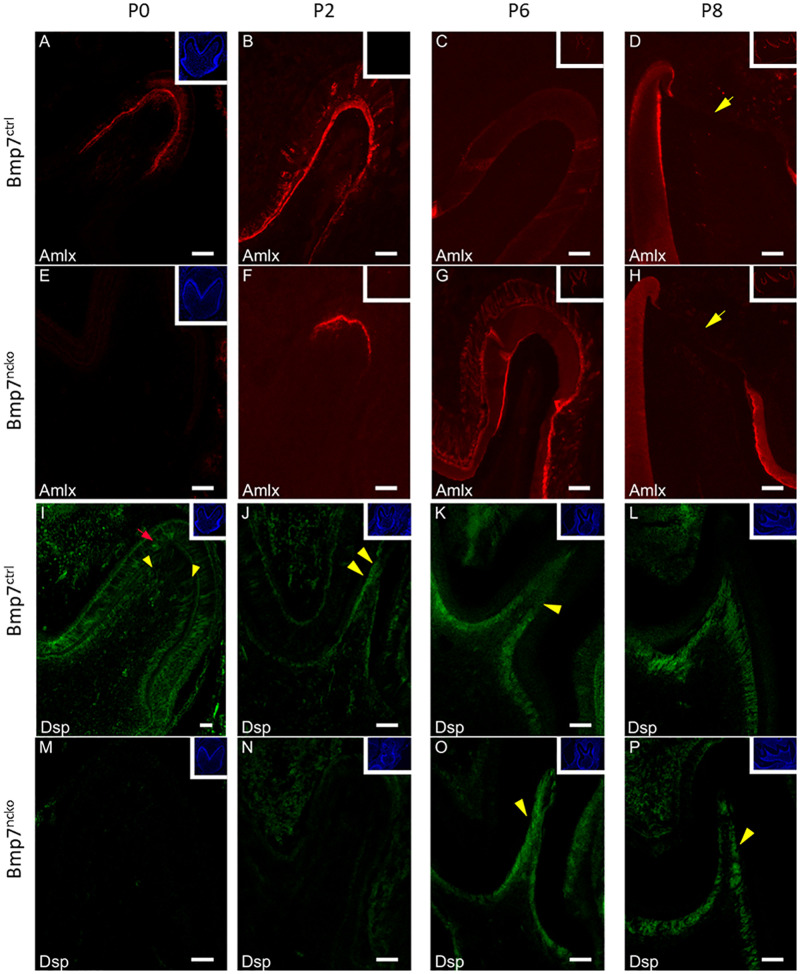
Bmp7 controls onset of dentin and enamel formation. Frontal sections of mandibular 1st molars at P0 **(A,E,I,M) (A–H)**, P2 **(B,F,J,N)**, P6 **(C,G,K,O)**, and P8 **(D,H,L,P)** from Bmp7 control **(A–D,I–L)** and Bmp7^ncko^ mice **(E–H,M–P)** stained for Amelogenin (Amlx) or Dentin sialoprotein (Dsp). For better orientation, DAPI stained sections are shown in the insert in the top left corner with the exception of **(C,D,G,H)**, where Amlx staining is shown. At P0 Amlx is absent and induction at tip of the cusp can be seen at P2 **(A,B,E,F)**. Overall expression pattern is conserved in the mutant evidenced by the presence of enamel-free zones [**(D,H)**, yellow arrow heads]. Similarly, Dsp expression is not initiated before P2 in the mutant **(N)**, while present in odontoblasts in control teeth from P0 onward [**(I,J)**, yellow arrowheads] or pre-ameloblasts (red arrow). Dsp expression at DN6 and DN8 **(K,L,O,P)** is comparable, although patchier staining can be observed in mutant teeth (yellow arrowheads).

We next confirmed whether the apparent delay in tooth mineralization was reflected on the molecular level by delayed expression of enamel and dentin matrix proteins. As shown in Figure 4, expression of both Amelogenin (Amlx) and Dentin sialoprotein (Dsp) was delayed by around 48 h in Bmp7-mutant teeth. In contrast to control teeth, where Amlx expression originated at the tip of the cusp, Amlx was completely absent in P0 Bmp7-mutant teeth (Figures 4A,E); 48 h later, at P2, when Amlx expression extended further down along the mineralization front in control teeth, some initial Amlx expression was now observed at the tip of the cusp in mutant teeth (Figures 4B,F). At subsequent stages (P6 and P8), Amlx expression became weaker at the tip of the cusps, where the most mature enamel would be found, but remained apparent more in cervical and in intercuspal areas in control teeth (Figures 4C,D). In the mutant, these local differences in Amlx expression were less apparent at P6. Expression toward cervical appeared to progress faster, while expression at the cusp tip remained (Figure 4G). At P8, expression between control and mutant teeth was comparable (Figures 4D,H). Similarly, Dsp expression was delayed by 48 h in Bmp7-mutant teeth. At P0, Dsp was expressed in control teeth in odontoblasts (Figure 4I, yellow arrowheads) and pre-ameloblasts (Figure 4I, red arrow) as expected. In contrast, Dsp was completely absent from mutant teeth (Figure 4M). At P2, Dsp expression in control teeth became more restricted to odontoblasts (Figure 4J, yellow arrowheads), whereas mutant teeth just started weakly expressing Dsp (Figure 4N). At P6 and P8, Dsp expression became largely comparable, although expression appeared to be less homogeneous in Bmp7 mutant teeth (Figures 4O,P, yellow arrowheads) when compared to the control (Figures 4K,L, yellow arrowhead). These findings establish that Bmp7 controls onset of tooth mineralization.

### Bmp7 Controls the Balance Between Canonical and Non-canonical Wnt Signaling

**FIGURE 5:**
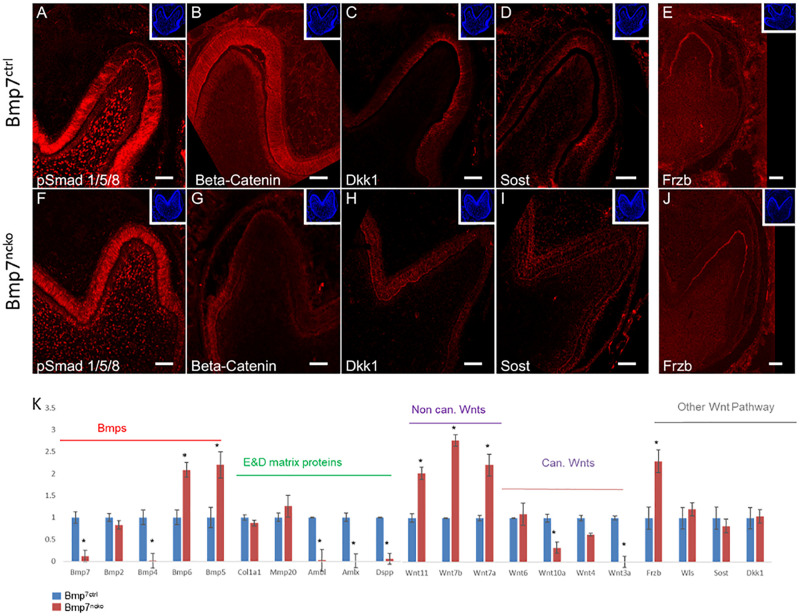
Bmp7 engages non-Smad1/5/8 signaling and controls the balance of canonical/non-canonical Wnt signaling. **(A–J)** Frontal sections of mandibular 1st molars from Bmp7 control **(A–E)** and Bmp7^ncko^ mice **(F–J)** at P0 stained for pSmad1/5/8 [Smad-dependent Bmp signaling, **(A,F)**], non-phosphorylated β-catenin [canonical Wnt signaling, **(B,G)**], Wnt antagonists Dkk1 **(C,H)**, Sost **(D,I)**, and Frzb **(E,J)**. Note persistence of pSmad1/5/8 in Bmp7^ncko^ teeth **(F)**, almost complete loss of non-phosphorylated β-catenin in the epithelium **(G)**, loss of polarization of Dkk1 **(H)**, and persistent expression of Frzb **(J)**. **(K)** RT-qPCR from RNA isolated from dissected 1st molars from Bmp7 control (blue) and Bmp7^ncko^ mice (red) showing changes to Bmp and Wnt signaling as well as selected enamel and dentin matrix genes. Note: lack of *Bmp4* expression and upregulation of *Bmp5* and *Bmp6*, downregulation of several canonical Wnt ligands (Wnts3a, 4, 10a) and up-regulation of non-canonical Wnts7a, 7b, 11, as well as lack of *Dspp, Amlx, Ambl* expression in Bmp7-mutant teeth. ^∗^*p* < = 0.05.

Bmp2 signals to pre-ameloblasts via Bmp-specific Smad1/5/8 signaling, which in turn regulates expression of the Wnt antagonists Sost and Dkk1 and odontoblast polarization (Malik et al., 2018). Loss of Bmp7 had no obvious effect on pSmad1/5/8 in the tooth epithelium (Figures 5A,F) indicating that Bmp7 might not engage canonical Bmp signaling or might only make a minor contribution to it. However, loss of Bmp7 led to an almost complete loss of canonical Wnt-signaling as seen by the very strong reduction of non-phosphorylated β-catenin in the epithelium of mutant teeth (Figures 5B,G). Loss of Bmp7 had no effect on the expression of the Wnt antagonists Dkk1 and Sost, but a lack of Dkk1 polarization was noted (Figures 5C,D,H,I). Expression of the Wnt-antagonist Frzb was unaffected and was expressed both in the control and mutant at the tip of the molar cusps (Figures 5E,J). We next assessed components of the Bmp and Wnt signaling pathways by RT-qPCR, as Bmp signaling is known to coordinate Wnt signaling during early tooth development (Itasaki and Hoppler, 2010) and loss of Bmp7 had an obvious effect on canonical Wnt signaling. As expected, expression of *Bmp7* was strongly reduced in mutant teeth. Expression of *Bmp2* was not affected, *Bmp4* was almost completely abolished, whereas both *Bmp5* and *Bmp6* were increased. *Bmp2,6*, and *7* are predominantly expressed in odontoblasts, whereas *Bmp4* and *5* show predominant epithelial expression (Supplementary Figure S2). We next tested for the expression of several Wnt ligands known to be expressed during tooth development (Sarkar and Sharpe, 1999; Suomalainen and Thesleff, 2010). The non-canonical Wnts *11, 7a, 7b* (Wnt/Ca^2 +^ and planar cell polarity signaling pathways) were all upregulated in Bmp7-mutant teeth, whereas several canonical Wnts engaging in β-catenin signaling were downregulated or almost absent (*Wnt3a, 4, 10)* (Figure 4K). Expression of *Dkk1, Sost* and *Wls* were unchanged, whereas *Frzb* was upregulated at the gene expression level (Figure 5K). We also tested several genes involved in the formation of the dentin or enamel matrix. *Col1* and *Mmp20* were unchanged in mutant teeth; however, the dentin-specific *Dentinsialophosphoprotein (Dspp)* and enamel-specific *Ameloblastin (Ambl)* and *Amelogenin (Amlx)* genes failed to be induced in Bmp7-mutant teeth (Figure 4K). This establishes that Bmp7 contributes to the complex, reciprocal mesenchymal–epithelial cross-talk to control the initiation of tooth mineralization.

### Initial Mineralization Delay Is Observed in All Molars and Results in Delayed Tooth Eruption

**FIGURE 6:**
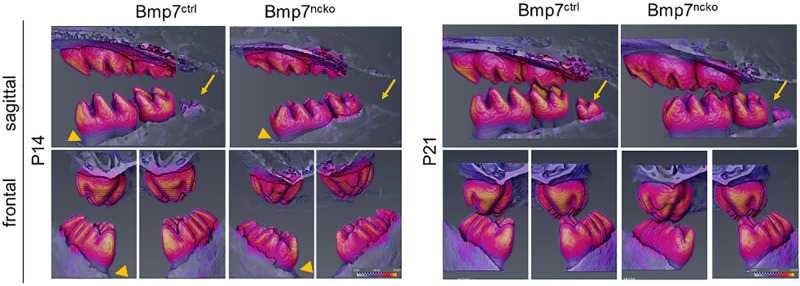
Delay in mineralization of 1st molar delays tooth eruption and maturation of the 3rd molar. μCT scans from P14 control and mutant mice reveal a delay in tooth eruption (arrowhead), a difference that is not evident at P21. The development of the 3rd molar is also delayed (arrows) evident as smaller size and reduced mineralization. Areas of highest radiodensity are also reduced in mutant teeth, as seen by less yellow in some of the cusps. This effect is also seen at P21.

**FIGURE 7:**
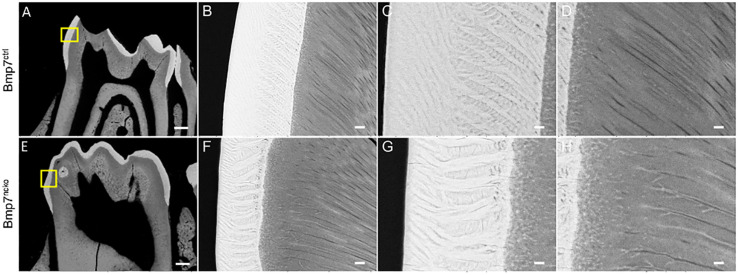
Loss of Bmp7 causes variations in enamel and dentin appearance. **(A,E)** Back scatter electron microscopy images of control and mutant P14 1st mandibular molars showing no gross anatomical differences in enamel/dentin structure. **(B–D,F–H)** High resolution view of control **(B,C,D)** and mutant **(F,G,H)** crowns suggests minor variations in organization of enamel and dentin bundles and the enamel–dentin junction. Scale bars: **(A,E)**: 200 μm; **(B,F,G)**: 12.5 μm; **(C,G,H)**: 5 μm.

We next tested whether tooth maturation was also delayed in the other molars and whether such a delay would be reflected in delayed tooth eruption. As can be seen on sagittal representations of μCT of P14 and P21 teeth, there was a clear delay in the maturation of the 3rd molar (Figure 5, arrows). Eruption of the 1st and 2nd molars was also delayed at P14 (Figure 6, arrowheads); however, this difference was less evident at P21. Furthermore, the radiodensity of control and mutant teeth appeared to be slightly different. Control teeth showed larger areas with the highest mineral density (Figure 6, yellow areas on cusps). This indicates that a delay in onset of tooth mineralization is reflected in an overall delay in dental age. Back scatter SEM analysis of adult control and mutant teeth revealed that loss of Bmp7 does not cause major changes to the mineralized structures (Figures 7A,E). The apparent loss of enamel free zones in the mutant tooth is not a general phenotypic feature, but because a more lateral tooth section was analyzed. Analysis of high power images showed differences in the orientation and thickness of enamel ribbons (Figures 7B,C,F,G). Dentinal tubules appeared also to be slightly altered, in particular in the region of the dentino–enamel junction (Figures 7D,H). This indicates that the changes to tooth mineralization caused by loss of Bmp7 are subtle and may not be clinically obvious.

## Discussion

A reduction in Bmp7 or loss of mesenchymal Bmp7 results in subtle changes to tooth size and cusp morphology and orientation, as well as the formation of extra cusps on the first upper and lower molars (Saito et al., 2016; Zurowski et al., 2018). Changes in attrition facets of Bmp7-mutant toothrows indicate concomitant changes in chewing behavior (Zurowski et al., 2018). Such changes could provide an advantage for accessing different food resources and thus contribute to niche diversification. Bmp signaling has long been proposed to contribute to the morphoregulation of teeth (Plikus et al., 2005), and expression patterns of various Bmps in tooth cusps support this notion (Meguro et al., 2019). Formation of molar cusps has been attributed to enamel knots, epithelial signaling centers that form at the tip of the cusp to coordinate proliferation and cytodifferentiation (Jernvall, 2000; Luukko et al., 2003). Lack of mesenchymal Bmp7 could either alter the distribution and timing of secondary/tertiary enamel knots or affect differential growth at the dentino–enamel junction, or both. Former would explain the appearance of additional cusps, and the latter occurs at the site where the main shape of the tooth is determined (Jernvall, 2000). Bmp7 expression correlated with lack of cell proliferation, and loss of Bmp7 resulted in continued proliferation in molar cusps providing an explanation for the larger tooth and wider cusp shape. As mineralization is not simultaneously initiated in all cusps, the increased duration in mesenchymal proliferation could allow for normally “hidden,” uninvolved secondary/tertiary enamel knots to become relevant. This could lead to the formation of additional cusps, in line with the finding that the temporal pattern of odontoblast terminal differentiation differs from enamel knot formation (Lisi et al., 2003). As the initiation of tooth mineralization goes in hand with an obligatory termination of odontoblast proliferation, controlling the time of mineralization onset provides an elegant means to fine-tune tooth size and cusp shape appearance.

Loss of mesenchymal Bmp7 results in an approximately 2-day delay in tooth mineralization. Thus, onset of tooth mineralization underlies an independent molecular control like other developmental events. It is not simply the consequence of a linear progression of a pre-determined developmental program. Loss of Bmp7 does not appear to alter the ordered sequence of tooth mineralization. Expression of Amlx and Dsp is still initiated at the tip of cusp and continues toward cervical as in control teeth. Although some cellular properties appear to be altered, such as lack of ameloblast polarization, the overall patterning of the cusp appears not to be changed. Frzb is still expressed at the molar cusp. This suggests that the morphological patterning, presumably driven by enamel knots, is not strictly dependent on concomitant cytodifferentiation of odontoblasts and ameloblasts, in line with earlier observations (Lisi et al., 2003).

The delay in mineralization is evident in all teeth investigated. First, the regulated temporal appearance of Bmp7 in pre-osteoblasts/osteoblasts just prior to the onset of mineralization is observed in all molars. Second, delayed mineralization of all molars was observed. Third, in the continuously growing incisor, the mineralization front was shifted to anterior (data not shown). Thus, the molecular control of tooth mineralization appears to be conserved within a toothrow and between different types of teeth. Apart from the reported morphological changes, the crowns of Bmp7^ncko^ teeth appear otherwise inconspicuous (Zurowski et al., 2018). However, local differences in maximal radiodensity were apparent and high resolution electron microscopy revealed additional minor differences in the appearance of enamel ribbons and the intercalation at the dentino–enamel junction. These discrete differences in mineralization could themselves contribute to altered wear facets in addition to the morphological differences (Zurowski et al., 2018). Thus, timing the onset of tooth mineralization is a novel parameter contributing to dental variation. It was somewhat surprising that the delay in mineralization would still be reflected by a delay in tooth eruption. One possibility is that the time of tooth eruption is directly linked to the onset of mineralization; however, it might be more plausible that mesenchymal Bmp7 in the pulp or alveolar bone has additional not yet understood functions that directly or indirectly affect tooth eruption. It is of interest to point toward the clonal appearance of Bmp7-positive cells observed in the first molar. This is reminiscent of nerve-associated glia cells that migrate and integrate into the odontoblast front (Kaukua et al., 2014). Whether Bmp7 can indeed be associated with the migration of such late migrating odontoblast precursors cells requires further validation. It certainly provides an interesting hypothesis to integrate molecular regulation of tooth mineralization with dental evolution.

**FIGURE 8:**
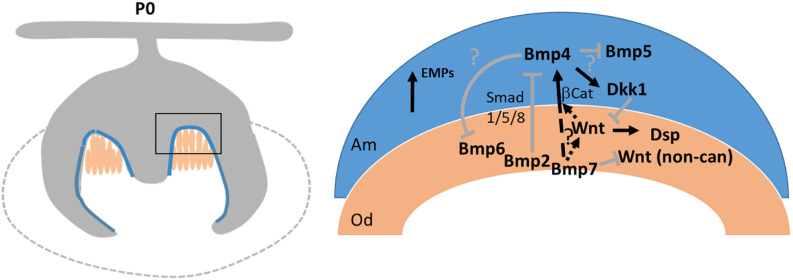
Schematic representation of how BMP signaling regulates the initial stages of tooth mineralization. **(Left)** Schematic of a molar at onset of mineralization. Blue: ameloblasts/pre-ameloblasts. Peach: odontoblasts. **(Right)** Intricate Bmp and Wnt signaling networks coordinate initiation of mineralization by engaging in multiple reciprocal mesenchymal-epithelial signaling events. Bmp7 signals independent of Bmp2 to regulate the balance between canonical/non-canonical Wnt signaling, induction of Bmp4 in ameloblasts, and polarization of Dkk1. Bmp6 expression is increased in the absence of Bmp7. Bmp2 is required for polarization of odontoblasts and downregulation of Dkk1 expression (Malik et al., 2018). Bmp4 expression in pre-ameloblasts/ameloblasts is increased in the absence of Bmp2. Loss of Bmp7 does not affect Bmp2 expression.

Several members of the Bmp family are expressed in odontoblast/ameloblasts around the time of tooth mineralization. We have previously shown that mesenchymal Bmp2 is required for directed deposition of the dentin matrix (Malik et al., 2018). Bmp2 is expressed concomitantly with Bmp7 at the onset of tooth mineralization. As summarized in Figure 8, Bmp2 engages in a mesenchymal-epithelial crosstalk, polarizes odontoblasts and signals ameloblasts in a Smad1/5/8-dependent manner to downregulate the expression of Dkk1 and Sost. This in turn enables canonical Wnt signaling important for the induction of Dsp expression in odontoblasts (Yamashiro et al., 2007). In contrast, Bmp7 either only makes a minor contribution to pSmad1/5/8 signaling - there is some residual pSmad1/5/8 activity in Bmp2-mutant teeth (Malik et al., 2018) – or it engages non-canonical Bmp signaling to directly or indirectly control canonical Wnt signaling in ameloblasts. Non-canonical Bmp signaling pathways include Mapk, Pi3K, and Tak signaling mediated via p38, pJun, or NFκB (Jun et al., 2010; Huang et al., 2014; Rodríguez-Carballo et al., 2016; Cui et al., 2019). None of those pathways is specific to Bmp signaling and all those pathways can be engaged by non-canonical WNT signaling. Signaling could occur in odontoblasts – its absence leading to delayed odontoblast maturation - or ameloblasts. While expression of canonical Wnt ligands is suppressed in Bmp7-mutant teeth, those engaged in non-canonical Wnt signaling (Tamura and Nemoto, 2016) are increased, complicating the detailed molecular dissection. Bmp7 could also affect canonical Wnt signaling directly. Expression of the β-catenin transcriptional partner Lef1 is controlled by Bmp7 in the dentate gyrus (Choe et al., 2013). Lef1 is under direct transcriptional control of Bmp4 (Armenteros et al., 2018), and Bmp4 expression was strongly reduced in Bmp7-mutant teeth. Overall, our data indicate that Bmp2 and Bmp7 are independently required to coordinate canonical Wnt signaling in odontoblasts. Our data furthermore indicate that canonical/non-canonical Wnt signaling serve different roles in the specification and differentiation of the molar cusp, reminiscent of Bmp/Wnt crosstalk during early tooth development (Yuan et al., 2015).

In addition to Bmp2 and Bmp7, several other Bmps are expressed in odontoblasts/ameloblasts around the time of onset of mineralization. Bmp6 is also predominantly expressed in the mesenchyme, and its expression increases in the absence of Bmp7. Bmp4 and Bmp5 are both expressed in the epithelium. Because of overlapping expression and promiscuous receptor binding, there might be functional redundancy, but combinatorial signaling or signaling as heterodimers is also considered (Yadin et al., 2016; Antebi et al., 2017; Kim et al., 2019). The Bmp family can be subdivided into several subfamilies. Bmp2 and Bmp4 are orthologs of *Drosophila* Decapentaplegic (Dpp), whereas Bmps 5/6/7/8 are orthologs of 60A or Glass-bottom-boat (Gbb) (Newfeld et al., 1999). Their evolutionary conservation argues against a general functional redundancy. In line with this, Bmp2 and Bmp7 exert very different and independent functions during early tooth mineralization mediated through differential engagement of downstream signaling events as shown here and previously (Malik et al., 2018). Loss of Bmp2 but not Bmp7 leads to an almost complete loss of pSMAD1/5/8 signaling in pre-ameloblasts, upregulation of BMP4 in the epithelium, persistence of polarized epithelial Dkk1, and upregulation of Sost in mesenchyme and epithelium. Loss of either signal results in the reduction of mesenchymal Wnt signaling activity, but only Bmp7 leads to an almost complete loss of canonical β-catenin signaling in pre-ameloblasts. The enamel/dentin phenotype in Bmp2 and Bmp7 mutants is also very different: whereas loss of mesenchymal Bmp2 leads to a dentinogenesis imperfecta-like phenotype, loss of mesenchymal Bmp7 shows only mild, inconspicuous changes to mineralization. Thus, heterodimer signaling does not make a major contribution as has recently been suggested for other developmental systems (Kim et al., 2019). A similar interplay between Bmps of the two subgroups might also take place in early ameloblasts, where concomitant expression of Bmp4 and Bmp5 was observed. Of interest, both Bmp2 and Bmp7 appear to regulate the expression of those two Bmps. Clearly, Bmp7 is an important factor controlling the induction of mineralization. However, mineralization also occurs in the absence of Bmp7. Mesenchymal Bmp6 was increased in the absence of Bmp7. We speculate that mesenchymal Bmp6 might play a similar role as Bmp7 and both can be used to fine-tune the timing of mineralization. It is tempting to speculate that Bmp7 might have taken over a more ancient role of Bmp6 to initiate tooth mineralization. For this to be the case, one would predict differential binding affinities of these two Bmps for either their cognate receptor or a regulatory Bmp antagonist. However, given the complexity of cusp shapes, state of mineralization (enamel, enamel-free zones), different timing of mineralization, it is equally likely that Bmp7 does not control mineralization in every single cusp and other molecular signals such as Bmp6 are also involved.

In summary, our findings show that regulation of onset of tooth mineralization is a hitherto unsuspected mechanism controlling tooth shape variation. Initiation of tooth mineralization is regulated by a complex epithelial–mesenchymal Bmp/Wnt-signaling network involving mesenchymal Bmp7. This network is separate and independent of the Bmp2-signaling network regulating odontoblast cell polarization. This unexpected complexity not only illustrates the multitude of cellular events required for tooth mineralization but also brings into question how successful the application of individual Bmp or Wnt signaling molecules can be to control odontoblast differentiation or initiate dentin repair. From an evolutionary perspective, addition of Bmp7 as initiator of tooth mineralization might be akin to an upgrade of an existing computer operating system. While not essential, it provides obviously sufficient advantage warranting its evolutionary incorporation. This notion provides an important and novel angle on the well-established concepts of gene redundancy and robustness of biological processes (Wagner, 2008): If biological processes evolve over time through addition of a novel functional layer while not completely removing the previous one, then what appears like redundancy might actually constitute reversion to a previous version of the same process.

## Data Availability Statement

The raw data supporting the conclusions of this article will be made available by the authors, without undue reservation.

## Ethics Statement

The animal study was reviewed and approved by the Research Ethics office of the University of Alberta (Animal Use and Care Committee protocol AUP1149).

## Author Contributions

ZM performed the experiments, analyzed the results, and wrote the manuscript. FE performed the experiments and analyzed the results. JT analyzed the results and edited the manuscript. DG conceived the study, performed the experiments, analyzed the results, wrote and edited the manuscript. All authors contributed to the article and approved the submitted version.

## Conflict of Interest

The authors declare that the research was conducted in the absence of any commercial or financial relationships that could be construed as a potential conflict of interest.
