# Supplementary material for: Mesenchymal Bmp7 Controls Onset of Tooth Mineralization: A Novel Way to Regulate Molar Cusp Shape
Source: Front Physiol. 2020 Jul 3;11:698. doi: 10.3389/fphys.2020.00698 (PMC7350786; doi:10.3389/fphys.2020.00698)
Supplement: Supplementary file 1 [file Data_Sheet_1.docx]

**Table S1: List of antibodies**

| **Primary Antibody** | **Supplier** | **Catalogue** | **Concentration** |
| --- | --- | --- | --- |
| DSP (mouse monoclonal clone 2C12.3) | Sigma | MABT37 | 1:200 |
| Amelogenin (Rabbit polyclonal) | Abcam | ab59705 | 1:200 |
| Phospho-Smad1/5/8 (Rabbit polyclonal) | Cell signaling | 13820 | 1:200 |
| Non-phospho β-catenin (Rabbit monoclonal) | Abcam | ab32572 | 1:200 |
| Dkk1 (Rabbit polyconal) | Abcam | ab63097 | 1:200 |
| Sost (Rabbit polyclonal) | Abcam | ab61034 | 1:200 |
| Frzb (Mouse monoclonal) | Santa Cruz  Biotechnology | sc-514350 | 1:200 |
|  |  |  |  |
| **Secondary Antibody** | **Supplier** | **Catalogue** | **Concentration** |
| Donkey anti-rat biotin | Southern Biotech | 6430-08 | 1:2000 |
| Donkey Anti Rabbit IgG (Alexa 647 conjugate) | Southern Biotech | A31573 | 1:1000 |
| Goat anti-Mouse IgG2b (Alexa 647 conjugate) | ThermoFisher | A-21242 | 1:1000 |

**Table S2: qPCR Primers**

| **Gene** | **Forward primer** | **Reverse primer** |
| --- | --- | --- |
| *Bmp2* | GGAAGACGTCCTCAGCGAAT | ACGGCTTCTTCGTGATGGAA |
| *Bmp4* | GAGCCAACACTGTGAGGAGT | ATACGGTGGAAGCCCTGTTC |
| *Bmp5* | AGATCTGGGATGGCAGGACT | GCTTGGGAACATGGTCTGGA |
| *Bmp6* | CATGAGCTTTGTGAACCTGG | CCCTCAGGAATCTGGGATAG |
| *Bmp7* | CGCCCATGTTCATGTTGGAC | ATGGTGGTATCGAGGGTGGA |
| *Wnt3a* | CACCACCGTCAGCAACAG | TCACTGCGAAAGCTACTCCA |
| *Wnt4* | GTTTCTCGCACGTCTCCTCT | CCTGCGACTCCTCGTCTTC |
| *Wnt6* | CTCCTACAGTGTGGTTGTCAGG | GCGCATCCATAAAGAGTCTTGA |
| *Wnt7a* | GGCTTCTCTTCGGTGGTAGC | TGAAACTGACACTCGTCCAGG |
| *Wnt7b* | CTTCACCTATGCCATCACGG | TGGTTGTAGTAGCCTTGCTTCT |
| *Wnt10a* | GCACTCTCTCGAAAACCTCG | CATGAGTGCCAGCATCAGTT |
| *Wnt11* | ATGCGTCTACACAACAGTGAAG | GTAGCGGGTCTTGAGGTCAG |
| *Frzb* | AGCCCGGATGACATAGTTGT | AGTACTGGACACTGCAGAGGG |
| *Wls* | TTTGGTGACATCCGACAGGG | CTTGCTTCCAGTACCCTGCA |
| *Sost* | TGTCAGGAAGCGGGTGTAGT | CCTCCTCCTGAGAACAACCA |
| *Dkk1* | CCGGGAACTACTGCAAAAAT | GTCAGTGTGGTTCTTCTGGGA |
| *Ambl* | CAAAGCATCCGCTTTTTACC | GCCTCCAAATCTTGGGAACAG |
| *Amlx* | TTTGCTATGCCCCTACCACCT | GTGATGAGGCTGAAGGGTGTG |
| *Dspp* | AGCAGTGAGGAAAACGGTGT | TGTTGCCTTTGTTGGGACCT |
| *Col1a1* | TGTCCCAACCCCCAAAGAC | CCCTCGACTCCTACATCTTCTGA |
| *Mmp20* | TGTGGAGTTCCTGATGTGGC | GACAGCTGTACTCCACGCAT |


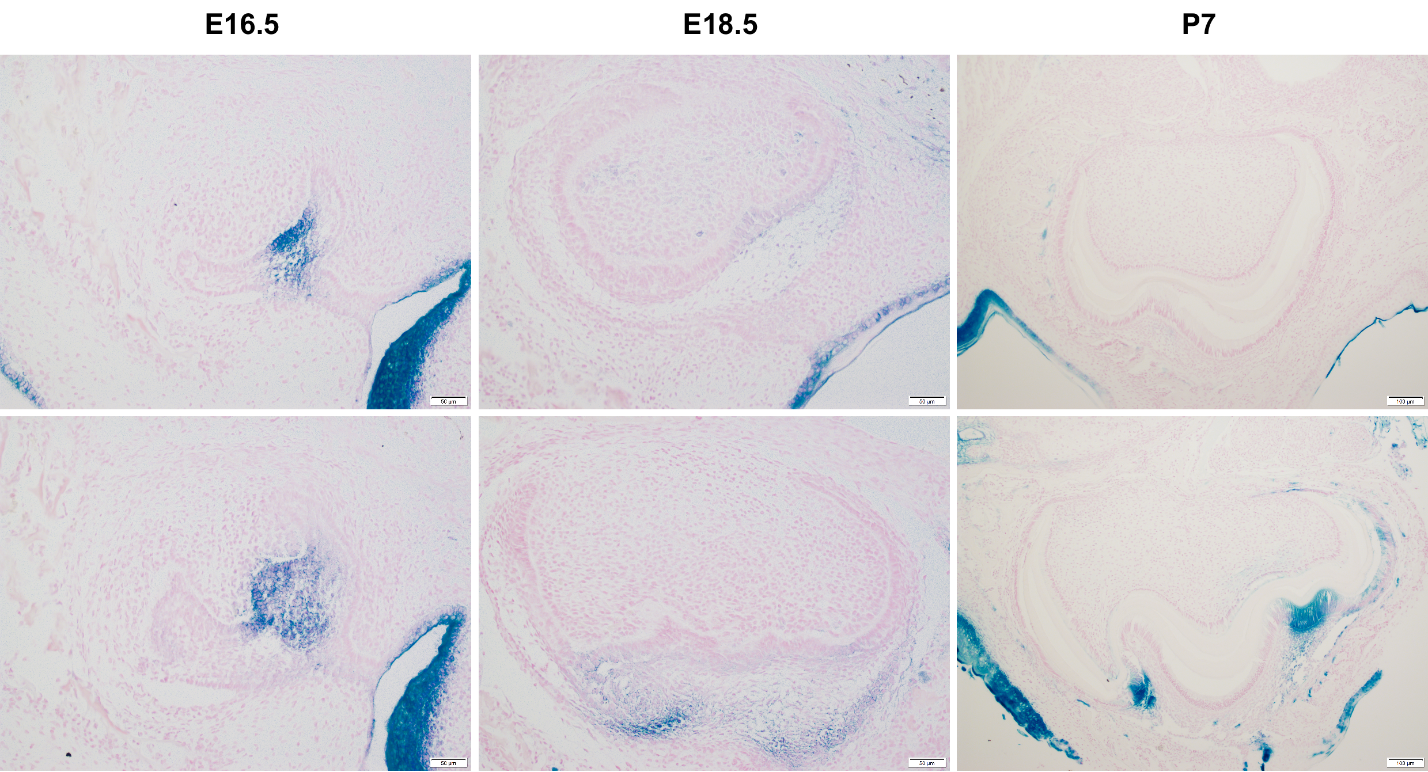
Figure S1:

**Figure 1: Supplemental *Bmp7lacZ* expression.** Frontal sections of molars from *Bmp7*LacZ reporter mice at E16.5, E18.5 and P7 illustrate Bmp7 expression in the enamel knot at E16.5, lack of mesenchymal expression at E18.5, as well as at P7 for comparison. All sections show strong epithelial expression for comparison.

Figure S2


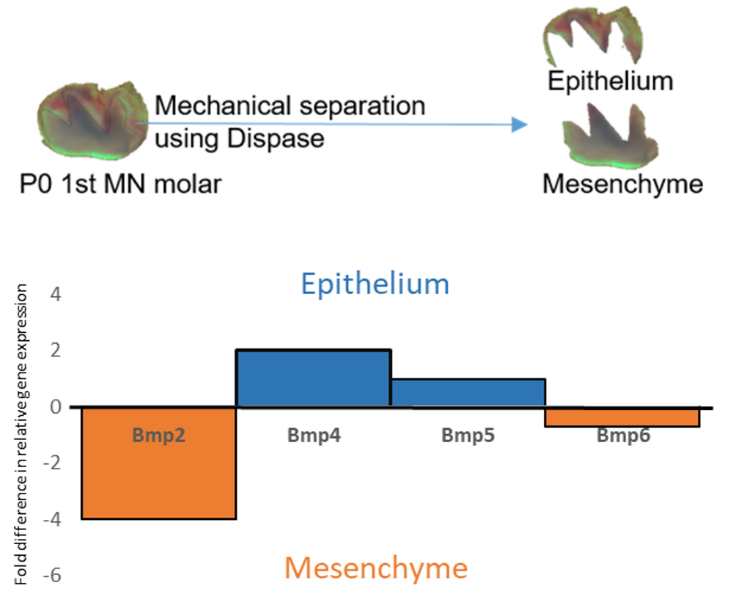


**Relative expression in epithelium and mesenchyme.** P0 mandibular molars were dissected, and epithelium was separated from the mesenchyme by enzymatic digestion. ΔCq RT-qPCR values were calculated and plotted to identify tissue of preferential expression.
